# Enhancing the solubility and antibacterial activity of novel molecular salts of enrofloxacin drug with isomeric pyridinedicarboxylic acids

**DOI:** 10.1038/s41598-024-80665-y

**Published:** 2024-11-26

**Authors:** Valeryia Hushcha, Anna Ben, Aleksandra Felczak, Katarzyna Lisowska, Zdzisław Kinart, Michał Gacki, Lilianna Chęcińska

**Affiliations:** 1 Faculty of Chemistry, University of Lodz, Pomorska 163/165, 90-236 Lodz, Poland; 2 University of Lodz Doctoral School of Exact and Natural Sciences, Narutowicza 68, 901-136 Lodz, Poland; 3 Faculty of Biology and Environmental Protection, University of Lodz, Banacha 12/16, 90-237 Lodz, Poland; 4 Institute of General and Ecological Chemistry, Faculty of Chemistry, Lodz University of Technology, Żeromskiego 116, 90-924 Lodz, Poland

**Keywords:** Antibacterial activity, Crystal engineering, Crystal structure, Enrofloxacin, Fluoroquinolones, Solubility, Structure-based drug design, Biomedical engineering

## Abstract

Enrofloxacin (EFX) is a third-generation synthetic fluoroquinolone with a broad spectrum of antibacterial activity but suffers from low water solubility, affecting its bioavailability. This study attempts to enhance the physicochemical and biological properties of enrofloxacin by converting it into multicomponent forms using crystal engineering concepts. Cocrystallization of enrofloxacin with isomeric pyridine-2,n-dicarboxylic acids (*n* = 3,4,5,6) resulted in four new crystalline salts (1:1): EFX·Py2,3DCA, EFX·Py2,4DCA, EFX·Py2,5DCA·H_2_O and EFX·Py2,6DCA·H_2_O; two of these are monohydrates. The protonation of the nitrogen atom of the piperazine moiety and the presence of crystallization water molecules were confirmed by single-crystal X-ray diffraction and Fourier transform infrared spectroscopy. Thermogravimetric analysis provided information on the thermal behaviour of multicomponent forms. The biological studies showed that the obtained salts are characterized by high antibacterial activity against Gram-positive and Gram-negative bacteria, and their haemolytic activity is low. The new salts demonstrate significantly greater solubility in water compared to the parent drug, along with enhanced antibacterial activity; hence, pyridinedicarboxylic acids appear to be efficient cocrystallizing agents for improving the efficacy of pharmaceutical ingredients.

**Supplementary Information:**

The online version contains supplementary material available at 10.1038/s41598-024-80665-y.

## Introduction

Quinolones have been the subject of considerable scientific and clinical interest since their discovery in the early 1960s^1,2^. They potentially offer many of the attributes of an ideal antibiotic, combining high potency, broad spectrum of activity, good bioavailability, and the potential for oral and intravenous formulations. In the decades since their discovery, the guidelines for their use have gradually become clearer and more efficient; however, heavy use, or overuse, has resulted in a considerable development of resistance that jeopardises the future of this entire class of antibiotics^3–5^.

The active structure of quinolones is based on a quinoline skeleton substituted by carboxylic group at position 3 and keto group at 4. Further modifications at different positions on the pharmacophore have provided a broader activity spectrum. The most groundbreaking discovery was the addition of a fluorine atom at position 6, which dramatically increased quinolone activity and gave rise to fluoroquinolone agents. The core skeleton was also modified by adding bulky nitrogen heterocycles (for example, the piperazine ring) at position 7 and the cyclopropyl group at position N1; both groups were found to be critical for biological activity^6,7^.

Fluoroquinolones are broad-spectrum antibacterial agents used in the treatment of bacterial infections^8–10^. The functional targets of fluoroquinolone antibiotics are DNA gyrase (type II topoisomerase) and topoisomerase IV, which participate in DNA replication; their interactions with DNA and their antibacterial activity, tested on various microorganisms, are of great importance and have been studied thoroughly^4,11–13^.

Enrofloxacin (EFX) (C_19_H_22_FN_3_O_3_, CAS No. 93106-60-6), 1-cyclopropyl-7-(4-ethyl-1-piperazinyl)-6-fluoro-1,4-dihydro-4-oxo-3-quinolinecarboxylic acid, is a typical synthetic third-generation fluoroquinolone and is characterized by high antibacterial activity and a broad spectrum of action. At low concentrations, enrofloxacin inhibits DNA replication and induces the SOS response, leading to inhibition of cell division, and has a reversible bacteriostatic effect. The bactericidal effect requires the use of high doses and is associated with chromosome fragmentation, which leads to rapid cell death. Enrofloxacin is active against Gram-positive and Gram-negative microorganisms, but not against anaerobic bacteria^14–16^. It is highly effective in the treatment of infections caused by *Staphylococcus aureus*, *Staphylococcus intermedius*, *Escherichia coli*, *Mannheimia haemolytica*, *Pseudomonas aeruginosa* and strains of *Streptoccocus* and *Salmonella*, thus it is widely used in veterinary medicine to cure respiratory, gastrointestinal, urinary and skin infections, as well as in the therapy of mastitis pigs, cows, calves and sheep^17–22^. Unfortunately, its broad spectrum of activity and good antibacterial properties have resulted in its widespread use for both therapeutic and prophylactic purposes. Therefore, enrofloxacin and its main metabolite ciprofloxacin have begun to appear in the natural environment at subinhibitory concentrations, contributing to the spread of fluoroquinolone antibiotic resistance.

Regarding the physicochemical properties of enrofloxacin, its water solubility is low^23^. Good solubility is an important requirement for ensuring optimal delivery, and is related to *inter alia* bioavailability, biopharmaceutical classification, and bioequivalence. Therefore, improving the physiochemical properties of drugs without changing their structural properties has become a hot topic in recent years. One of the most effective ways to improve the solubility and bioavailability of poorly-soluble drugs is to convert them into salts^24,25^. Salt preparation depends primarily on the acid-base property between the active pharmaceutical ingredient (API) and the cocrystallizing substance (coformer). Completely ionised components can easily form salts when their acid-base difference is relatively large (ΔpKa > 4)^26^.

**Fig. 1:**
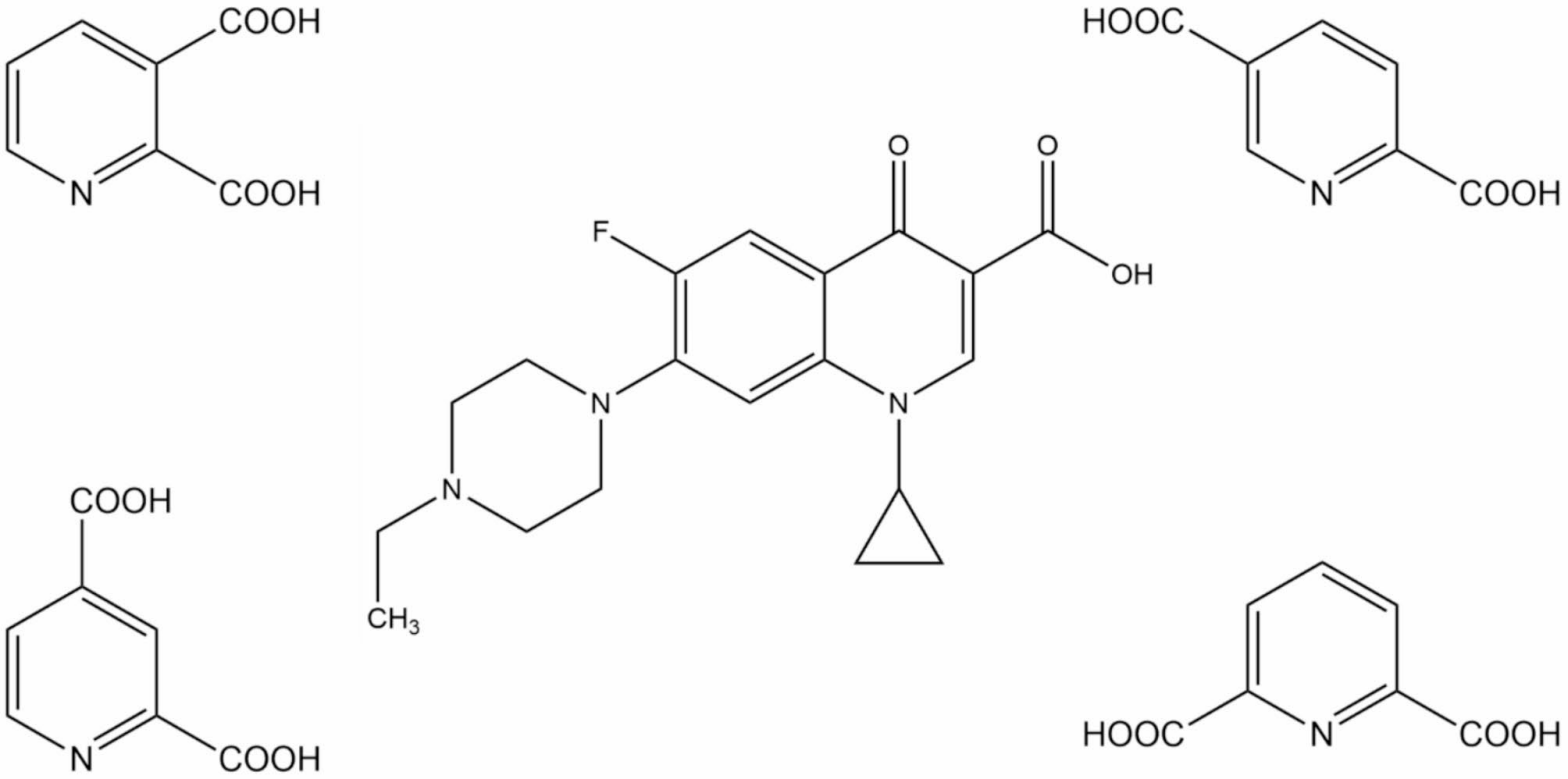
Structures of enrofloxacin and isomeric pyridine-2,n-dicarboxylic acids (*n* = 3,4,5,6).

The present study uses crystal engineering concepts^27,28^ to obtain enrofloxacin salts with pyridine-2,*n*-dicarboxylic acids (*n* = 3,4,5,6) (Fig. 1). The selected acids appear to be promising coformers considering the ΔpKa rule and their quite good water solubility, however they are not generally regarded as safe (GRAS) substances according to the FDA (U.S. Food and Drug Administration) recommendations^27^. Generally, pyridine-based heterocycles are one of the most widely-used pharmacophores in the field of drug development due to their better absorption quality and fewer side effects than those based on benzene ring^28–29^ Pyridinedicarboxylic acids containing a pyridine ring and two carboxylic groups can provide various coordination modes; they are therefore widely used for the construction of coordination compounds with extensive applications in medicine^30^. Our study shows that pyridinedicarboxylic acids can also play a valuable role as coformers in cocrystallization with an active substance, to enhance its physicochemical and biological properties in multicomponent forms.

A search for multicomponent forms of enrofloxacin in the Cambridge Structural Database (CSD version 5.45, June 2024^31^) resulted in 27 hits^32–42^, of which 21 appeared to be molecular salts, one cocrystal and 5 salt cocrystals (or ionic cocrystals); unpublished private communications were excluded from the investigation. Interestingly, most of them (16 crystals) are hydrates and/or solvates. The coformers used in the studies are small organic aliphatic acids (e.g., adipic, oxalic, pimelic, succinic) or aromatic acids (e.g., picric, 3,5-dihydroxybenzoic, 2,6-dihydroxybenzoic) or amines (tyramine, ethanolamine). Nicotinic acid is the only reported heterocyclic coformer combined with enrofloxacin^36^. The reported studies mainly consider the structural aspects of multicomponent forms of EFX and improvements in their physicochemical properties, such as solubility in water or stability; only one report presents the antibacterial activity of enrofloxacin with *p*-nitrobenzoic acid (salt cocrystal) against *E. coli*, *S. aureus* and *S. typhi* compared to pure EFX by the filter paper method^39^.

In the present study, four new salts of enrofloxacin with isomeric pyridinedicarboxylic acids are tested against two Gram-positive strains (*S aureus*, *S. pyogenes*) and two Gram-negative bacteria (*E. coli*, *P. aeruginosa*) using microdilution method; toxicity tests with red blood cells are also performed.

## Results and discussion

### Structural characterization

**Fig. 2:**
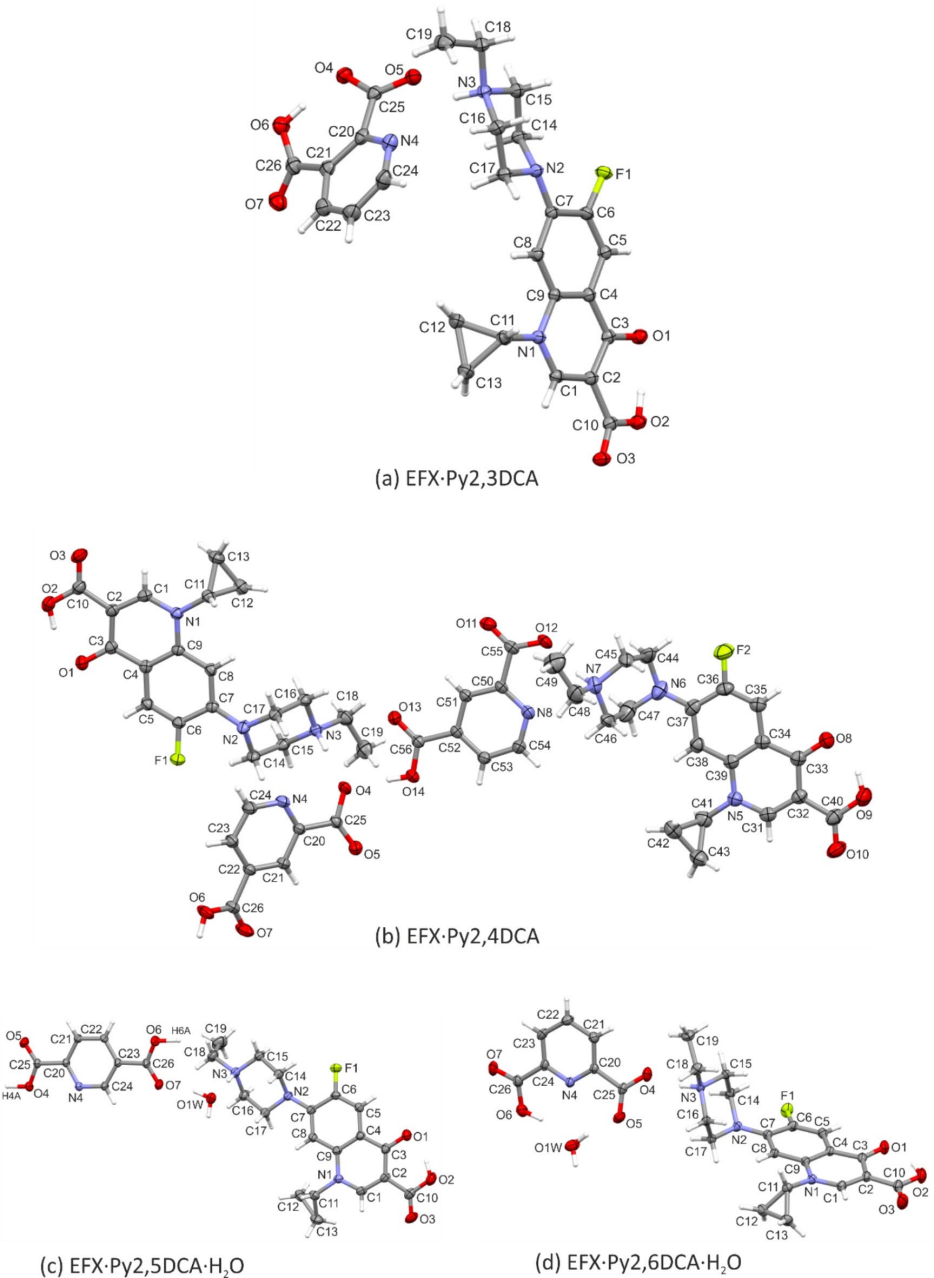
View of the asymmetric unit of EFX·Py2,3DCA (**a**), EFX·Py2,4DCA (**b**), EFX·Py2,5DCA·H_2_O (**c**) and EFX·Py2,6DCA·H_2_O (**d**), with atom-numbering schemes. Displacement ellipsoids are drawn at the 50% (**a**) and 30% (**b**–**d**) probability level. H-atoms are shown as spheres of arbitrary radii.

Figure 2a–d show the asymmetric unit of the four novel salts of enrofloxacin (EFX) with pyridine dicarboxylic acids, namely pyridine-2,3-dicarboxylic acid (Py2,3DCA), pyridine-2,4-dicarboxylic acid (Py2,4DCA), pyridine-2,5-dicarboxylic acid (Py2,5DCA) and pyridine-2,6-dicarboxylic acid (Py2,6DCA). Two salts crystallize as a stoichiometric monohydrate, EFX·Py2,5DCA·H_2_O and EFX·Py2,6DCA·H_2_O, in which the water molecule is firmly embedded in the overall hydrogen-bonded network (Section “Supramolecular architecture”). In case of EFX·Py2,4DCA, the asymmetric unit consists of two cation-anion pairs.

In all salt structures, the enrofloxacin molecule is a cation. The formal positive charge is located on the protonated nitrogen atom (N3/N7) of the piperazine ring.

The bicyclic core consists of a heterocyclic ring (ring 1) condensed with a benzene ring (ring 2). The dihedral angle between the least-squares planes of these rings does not exceed 4° (Table S1 in the supplementary information); thus the 10-membered quinoline fragment can be considered as planar.

The crystals of the analysed salts are racemic; the enrofloxacin molecules are conformationally chiral. In the asymmetric unit, they were chosen in a conformation in which the cyclopropyl group is orientated forward relative to the quinolone core; exceptionally, this substituent is inverted in a second independent molecule (B) in EFX·Py2,4DCA (Fig. S1 in the supplementary information).

Additionally, although the piperazine ring adopts a chair conformation, two opposite chair forms can be indicated from the ring puckering parameters^43^; a θ value close to 0 was calculated for molecule A in EFX·Py2,4DCA and EFX·Py2,5DCA as contrary to θ value close to 180° in the remaining cases (Table S2 in the supplementary information). In this way, the mutual arrangement of the quinolone core and the piperazine ring differentiates the enrofloxacin molecules compared in the asymmetric unit of four salts (Fig. S1 in the supplementary information).

The observed molecular differences can also be confirmed by the dihedral angle between the best planes of the benzene and piperazine rings (Table S1 in the supplementary information), or the angle between the N2−C7 (or N6−C37) bond and the normal to the Cremer & Pople mean plane of the piperazine ring (Table S3). The latter angular parameter indicates that the quinolone moiety occupies equatorial site in the piperazine ring in EFX·Py2,3DCA and EFX·Py2,5DCA·H_2_O, bisectional one in EFX·Py2,4DCA and axial one in EFX·Py2,6DCA·H_2_O.

Interestingly, regardless of the molecular conformation of the EFX, the intramolecular C—H···F hydrogen bond is formed in all its molecular structures reported herein. To allow comparison, the position of the (C)−H atom was normalized to correspond to neutron diffraction data: C−H = 1.10Å according to Spek (2020)^44^. The H···F distances are in the range 1.97–2.13 Å and C—H···F angles vary from 121° to 129° (Table S4). Theoretical energy calculations proposed by Karanam & Choudhury^34^ showed that an increase in the proton···acceptor distance up to 3.64Å increases the energy of the EFX molecule, indicating that the intramolecular C—H···F interaction provides additional stability to the EFX molecule.

Another important interaction stabilizing the EFX molecule is an intramolecular O—H···O hydrogen bond between the carboxylic group and the carbonyl oxygen atom of the quinolone moiety; this allows the formation of the six-membered synthon S(6)^45,46^. This interaction is typical for neutral and cationic forms of fluoroquinolones^34,40^. After normalization (O−H = 0.98Å) (Table S5), the H···O distances are in the range 1.55–1.64 Å, and the O—H···O angles vary from 149° to 159° showing that the hydrogen bond is much stronger than the C−H···F interaction mentioned above so a higher impact on the stability of the EFX molecule is expected. An intramolecular O6—H6*A*···O4 hydrogen bond was also found for the Py2,3DCA anion (Tables S5–S6 in the supplementary information); as it is characteristic for a crystal structure of pyridine-2,3-dicarboxylic acid itself^47^.

### Supramolecular architecture

In all compounds obtained, one of the two acidic protons of the pyridinedicarboxylic acids had transferred to the nitrogen atom of the piperazine ring (N3/N7) of the enrofloxacin molecule, thus forming the molecular salt. For EFX·Py2,3DCA, EFX·Py2,4DCA and EFX·Py2,6DCA·H_2_O, geometric parameters (Table S7 in the supplementary information) confirm that the proton from the carboxylic group (at position 2), nearest to the pyridine nitrogen atom, preferably transfers to the EFX molecule. For the carboxylic group, the single C−O(H) bond lengths range from 1.2952(2)Å in EFX·Py2,3DCA to 1.3195(2)Å in EFX·Py2,6DCA·H_2_O, while the double C = O bond lengths vary from 1.2094(2)Å in EFX·Py2,6DCA·H_2_O to 1.2248(2)Å in EFX·Py2,3DCA. For the deprotonated carboxylate group in EFX·Py2,3DCA and EFX·Py2,4DCA the single C−O(-) bond lengths are reasonably shorter than the C−O(H); they range from 1.278(3)Å to 1.290(2)Å. In the EFX·Py2,6DCA·H_2_O salt, the negative charge of the carboxylate group is exceptionally delocalized, which is confirmed by less varied C−O distances, 1.2354(2)Å and 1.2468(2)Å. The situation in the structure of EFX·Py2,5DCA·H_2_O is different from the others due to the equal sharing of hydrogen atoms between neighboring acid molecules.

The supramolecular architecture of all new EFX forms is mainly determined by O−H···O and C−H···O interactions; this study includes only the most structurally-significant interactions, these being hydrogen bonds with D−H···A angles greater than 120°, and proton···acceptor distances 0.20 Å shorter than the sum of the van der Waals radii of the interacting atoms (Table S6 in the supplementary information).

**Fig. 3:**
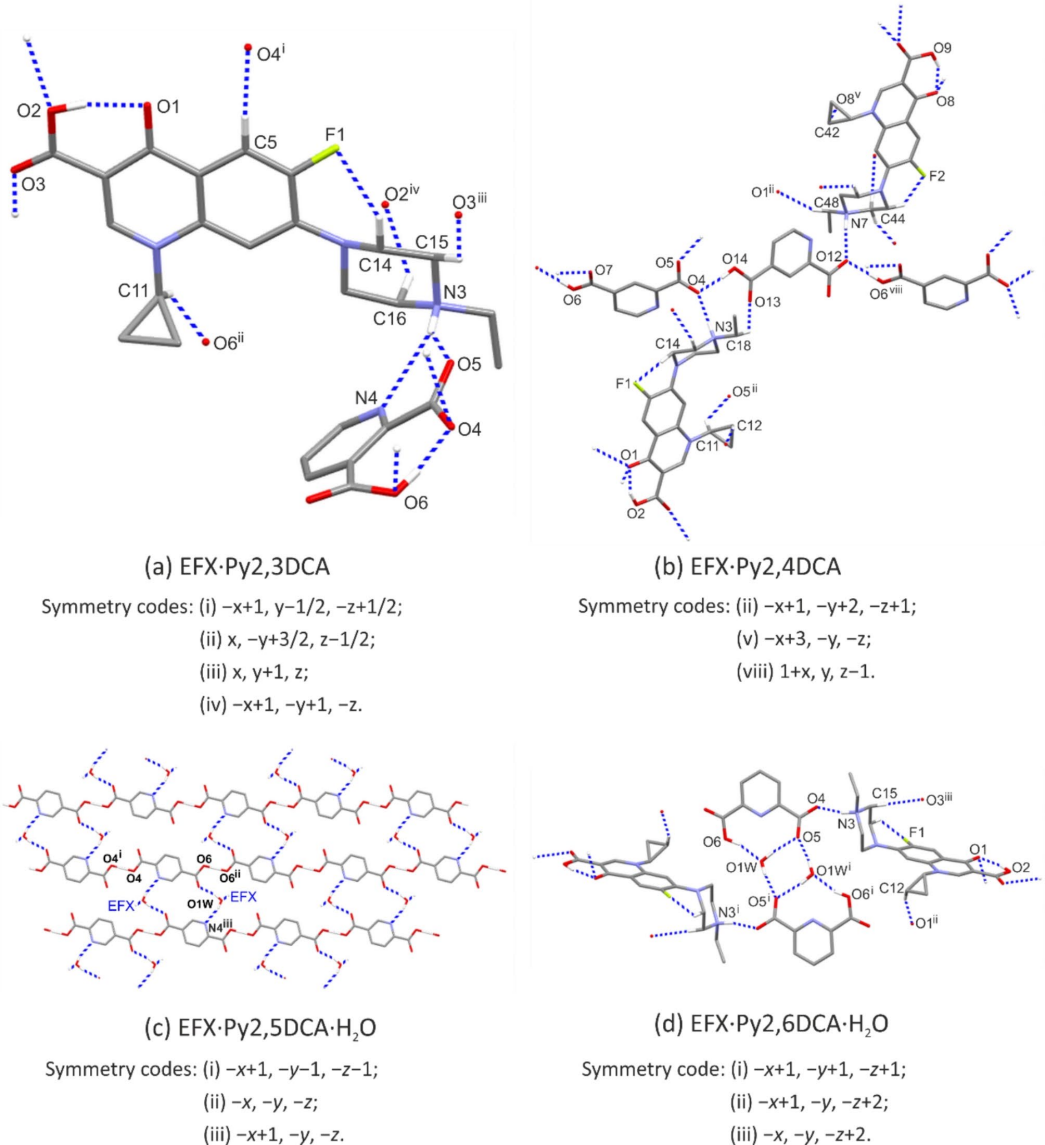
A scheme of intermolecular interactions for EFX·Py2,3DCA (**a**) EFX·Py2,4DCA (**b**) EFX·Py2,5DCA·H_2_O (**c**) and EFX·Py2,6DCA·H_2_O (**d**).

In the EFX·Py2,3DCA salt, the ionic components of the asymmetric unit are linked by two hydrogen bonds N3−H3*A*···O5 and N3—H3*A*···N4, forming the $\:{R}_{1}^{2}\left(5\right)$ ring motif (Fig. 3a). The C15−H15*B*···O3(*x*, *y* + 1, *z*) hydrogen bond propagates the enrofloxacin cations along the crystallographic *b* axis whereas C16−H16*B*···O2(− *x* + 1, −*y* + 1, −*z*) generates the ring motif between two centrosymmetrically-related EFX cations. Furthermore, two interactions with an anion, C5−H5···O4(− *x* + 1, *y* − 1/2, −*z* + 1/2) and C11—H11···O6 (*x*, −*y* + 3/2, *z* − 1/2), allow EFX dimers to be repeated along the *c* axis. Finally, supramolecular di-periodic complex sheets (100) are formed (Fig. S2a in the supplementary information). The sheets are connected to each other *via* the aromatic π-π stacking interactions between outermost pyridine rings of anions (Table S8 in the supplementary information).

In EFX·Py2,4DCA salt, the pyridine anions are linked through the O14−H14···O4 and O6−H6···O12(*x* − 1, *y*, *z* + 1) hydrogen bonds to form a mono-periodic chain motif running parallel to the [10$\:\stackrel{-}{1}$] direction. Both acceptor oxygen atoms, O4 and O12, are also acceptors of the N3−H3*A*···O4 and N7−H7*A*···O12 hydrogen bonds from the enrofloxacin cation (Fig. 3b). Numerous C−H···O interactions extend the supramolecular structure of EFX·Py2,4DCA to a tri-periodic assembly (Fig. S2b in the supplementary information).

In the EFX·Py2,5DCA·H_2_O salt, despite the transfer of one proton to the enrofloxacin molecule, both carboxyl groups of Py2,5DCA remain partially protonated because the second hydrogen atom is shared equally between the two acidic groups; the anion is formally monocarboxylate. The H4*A* and H6*A* hydrogen atoms lie at special positions on the centres of symmetry at (0.5, -0.5, -0.5) and (0, 0, 0), exactly halfway between the two oxygen atoms (the O−H and H···O distances are both 1.23Å). Two O4−H4*A*···O4(− *x* + 1, −*y* − 1, −*z* − 1) and O6−H6*A*···O6(− *x*, −*y*, −*z*) hydrogen bonds create mono-periodic polymeric chains built from anions. The adjacent chains are linked each other by water molecules through the O1*W*−H1*W*1···O7 and O1*W*−H2*W*1···N4(− *x* + 1, −*y*, −*z*) hydrogen bonds, thus forming di-periodic sheets (01$\:\stackrel{-}{1}$) (Fig. 3c). Enrofloxacin cations attach to these sheets *via* the N3−H3*A*···O1*W* hydrogen bond. In EFX·Py2,5DCA·H_2_O, no cation-anion pair is formed; in this case, the water molecule acts as an acceptor. The space between sheets composed of anions and water molecules provide room for enrofloxacin cations to interact with each other. The C15−H15*A*···O3(*x* − 1, *y* − 1, *z* − 1) hydrogen bond is responsible for a chain motif whereas the C16−H16*A*···O2(− *x* + 1, −*y* + 2, −*z* + 1) interaction generates centrosymmetric dimers, thus generating a tri-periodic supramolecular structure (Fig. S2c in the supplementary information).

In the EFX·Py2,6DCA·H_2_O salt, the Py2,6DCA anion interacts with the water molecule *via* O6−H6*A*···O1*W.* The water molecule donates two hydrogen bonds, O1*W*−H2*W*1···O5 and O1*W*−H1*W*1···O5(− *x* + 1, −*y* + 1, −*z* + 1). This creates a four-molecule centrosymmetric finite pattern with three condensed hydrogen-bonded ring motifs, to which the enrofloxacin cations are attached *via* the N3—H3*A*···O4 hydrogen bond (Fig. 3d). The EFX cations interact each other through the C12—H12*A*···O1(− *x* + 1, −*y*, −*z* + 2) and C15—H15*A*···O3(− *x*, −*y*, −*z* + 2) hydrogen bonds, forming infinite chains of fused centrosymmetric rings running parallel to the [100] direction. Finally, the chain-of-rings motifs and finite patterns are combined into di-periodic assemblies (0 2 1) (Fig. S2d in the supplementary information); which can be further arranged in a tri-periodic network by the aromatic π-π stacking interactions between pyridine rings of anions.

### Spectroscopic analysis

**Fig. 4:**
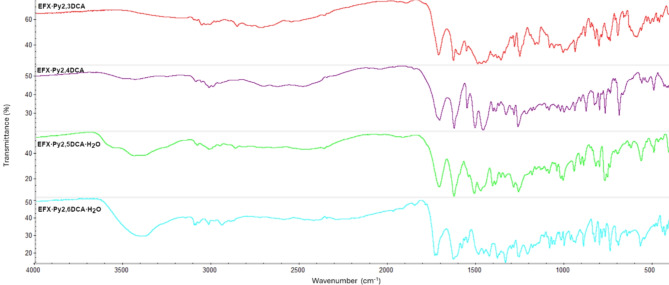
Fourier-transform infrared spectra of EFX·Py2,3DCA (red), EFX·Py2,4DCA (violet) EFX·Py2,5DCA·H_2_O (green) and EFX·Py2,6DCA·H_2_O (cyan).

Fourier-transform infrared (FT-IR) spectra for all four salts of enrofloxacin are shown in Fig. 4 (and Figs. S4–S7 in the supplementary information). The FT-IR spectrum of EFX (Fig. S3) reveals characteristic absorption bands at 1288 cm^− 1^ (C−F band), 1506 cm^− 1^ (stretching vibration peak of the C$\:=$C bond), 1627 cm^−1^ (C$\:=$O stretching vibration in the carbonyl group) and 1736 cm^− 1^ (C$\:=$O stretching vibration in the carboxyl group)^41^.

The FT-IR spectra for pyridine-2,n-dicarboxylic acids (*n* = 3,4,5,6) show characteristic bands at the range of 1693 cm^− 1^ to 1728 cm^− 1^ assigned to C$\:=$O stretching vibration. The absorption bands appearing in the range of 1574 cm^−1^ to 1608 cm^− 1^ are related to C−N. Around 3100 cm^− 1^ characteristic peaks are observed due to O−H stretching vibration in the acid molecules.

After the formation of the analysed salts, EFX·Py2,3DCA, EFX·Py2,4DCA, EFX·Py2,5DCA·H_2_O and EFX·Py2,6DCA·H_2_O, the C$\:=$O stretching vibration in the carboxyl group shifted to 1716 cm^−1^, 1709 cm^− 1^, 1712 cm^−1^ and 1735 cm^− 1^, while the C$\:=$O stretching vibration in the carbonyl group shifted slightly to 1630 cm^− 1^, 1627 cm^−1^, 1628 cm^− 1^ and 1631 cm^− 1^, respectively. Bands also formed in the range of 2600 cm^− 1^ – 3000 cm^− 1^ due to protonation of the piperazine ring in the EFX molecule. The broad bands observed at 3435 cm^− 1^ and 3393 cm^− 1^ in EFX·Py2,5DCA·H_2_O and EFX·Py2,6DCA·H_2_O are attributed to the stretching of the O−H bonds of the water molecule.

### Thermal analysis

**Fig. 5:**
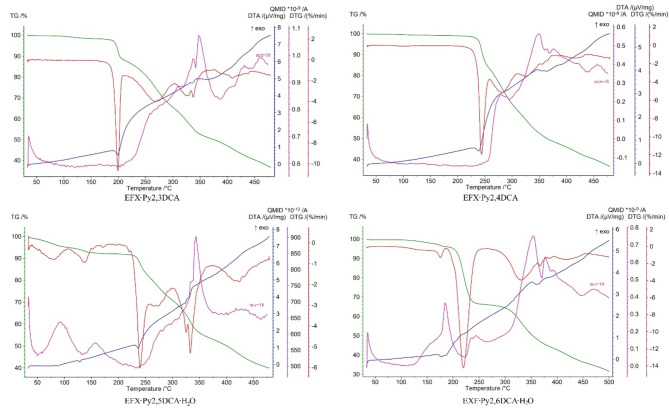
Thermoanalytical curves for the analysed enrofloxacin salts with pyridinedicarboxylic acids and QMID (quasi multiple ion detection) for H_2_O^+^ ion current (m/z = 18) at 25–500 °C. The TG (thermogravimetric) curves are plotted in green, DTG (derivative thermogravimetric) in red, DTA (differential thermal analysis) in blue and QMID in pink.

The thermal decompositions of analysed enrofloxacin salts are shown in Fig. 5 (and Fig. S8 in the supplementary information). Recorded data indicate that the EFX·Py2,3DCA and EFX·Py2,4DCA salts are anhydrous, while EFX·Py2,5DCA·H_2_O and EFX·Py2,6DCA·H_2_O are hydrates. The latter two start to decompose by dehydration, which proceeds in two steps for EFX·Py2,5DCA·H_2_O or a single one for EFX·Py2,6DCA·H_2_O. In EFX·Py2,5DCA·H_2_O, the first step is represented by a loss of adsorbed water (4.43%) at 35–100 °C, while the second is attributed to the release of crystallization water at 100–165 °C (mass loss 2.92%; theoretical 3.31%). Those steps are represented as two distinct endothermic peaks at 90 °C and 130 °C on the DTA curve. Additionally, the maximum ion current signals assigned to H_2_O^+^ (m/z = 18, QMID curve) are observed at 90 °C and 155 °C.

In EFX·Py2,6DCA·H_2_O, the loss of crystallization water molecules proceeds at 105–190 °C (mass loss exp. 3.50% and calc. 3.31%), as confirmed by characteristic H_2_O^+^ ion current signals with the maximum at 180 °C. The corresponding endo-effect is visible on the DTA (differential thermal analysis) curve at 175 °C. Thermal destruction of anhydrous compounds begins after 180 °C (EFX·Py2,3DCA), 220 °C (EFX·Py2,4DCA), 200 °C (EFX·Py2,5DCA·H_2_O) and 190 °C (EFX·Py2,6DCA·H_2_O), respectively. The latter transformations are represented by a three-step process for each compound. The characteristic exo- and endothermic effects are visible on the DTA curves. All salts decompose completely up to 700 °C.

Regarding the relationship between the thermal decomposition of enrofloxacin salts, their supramolecular architectures and their corresponding packing index^44^, it can be seen that the tri-periodic supramolecular structures have a lower packing index, e.g. PI(EFX·Py2,4DCA) = 69.6% and PI(EFX·Py2,5DCA·H_2_O) = 71.0%, than those that form primarily di-periodic structures, and which are further organized in tri-periodic networks by π-π stacking interaction, e.g. PI(EFX·Py2,6DCA·H_2_O; EFX·Py2,3DCA) = 72.1%. In addition, the former are characterized by higher decomposition temperatures (220 °C and 200 °C vs. 190 °C and 180 °C).

### Solubility studies

As the parent drugs are often poorly soluble, one of the most desirable physicochemical property for multicomponent forms of active substances is water solubility. The solubility of enrofloxacin in water ranges from 0.14 mg/mL^41^ to 0.61 mg/mL^34^ depending on the method and the measurement conditions. In the present study, the solubility of commercially-available EFX in water was measured as 0.176 mg/mL at 25 °C by the gravimetric method^48^, while those of its crystalline salts are 6.387 mg/mL (EFX·Py2,3DCA), 20.325 mg/mL (EFX·Py2,4DCA), 3.013 mg/mL (EFX·Py2,5DCA·H_2_O) and 17.552 mg/mL (EFX·Py2,6DCA·H_2_O); these values are approximately 36, 115, 17 and 100 times greater than those of EFX, respectively.

The best dissolution enhancers are the acid coformers Py2,4DCA and Py2,6DCA; however, it is difficult to identify a suitable property responsible for differentiation of solubility among the four tested isomeric pyridinedicarboxylic acids. However, the lowest solubility in water of pure Py2,5DCA (1.237 mg/mL at 25 °C) seems to be related with the lowest increase in solubility for its enrofloxacin salt; in contrast, the remaining isomeric acids demonstrate similar solubility in water (about 5 mg/mL), but their salts no longer behave similarly.

Recent studies on multicomponent forms of enrofloxacin showed that other coformers demonstrate a significant increase of solubility in water compared to the parent drug: for example, succinic acid (20.65 mg/mL), oxalic acid (16.21 mg/mL) acetic acid (highly soluble)^34^, malic acid (16.13 mg/mL) and adipic acid (41.06 mg/mL)^41^.

### Assessment of antibacterial activity

Enrofloxacin, is characterized by high antimicrobial activity and a broad spectrum of action, and is one of the key antibiotics used in veterinary medicine; however, it must reach the appropriate concentrations in tissues to produce the desired effect, and its accumulation is hampered by the fact that it is a lipophilic compound characterized by low solubility in water. The bioavailability of enrofloxacin varies according to the method of administration and the animal, as well as the presence of other ions or the addition of lipophilic substances^16^. Therefore, it is so important to synthesize and characterize its new multicomponent forms e.g. salts, which have better solubility than enrofloxacin but retain its high antibacterial activity. The increase in solubility, in turn, has a direct impact on the bioavailability of the drug and the doses that are used in therapy.

This study used isomeric pyridinedicarboxylic acids as cocrystallizing agents to prepare the salts of enrofloxacin. These new multicomponent forms and pure (commercial) enrofloxacin were subjected to biological testing. All tested compounds and pure enrofloxacin demonstrated a minimal bactericidal concentration (MBC) of 0.25 mg/L against *S. aureus* (Table 1) and MIC value of 0.0078 mg/L in cultures containing *E. coli* (Table 1). It is worth noting that the tested salts have a higher molar mass than the parent substance and contain approximately 66–68% of pure enrofloxacin, resulting in a lower content of the active pharmaceutical ingredient. In summary, the obtained salts retain the antimicrobial properties of the parent drug, or exhibited even better antibacterial activity, effectively inhibiting the growth of both Gram-positive and Gram-negative microorganisms. A similar effect was achieved by Liu et al.,^49^ who obtained salts/cocrystals of enoxacin and dicarboxylic acids, which inhibited the growth of *S. aureus*, *S. albus* and *E. coli*, while also exhibiting greater solubility.

**Table 1:** Antibacterial activity of enrofloxacin salts.

	EFX		EFX·Py2,3DCA		EFX·Py2,4DCA		EFX·Py2,5DCA ·H_2_O		EFX·Py2,6DCA ·H_2_O	
Strain	MIC	MBC	MIC	MBC	MIC	MBC	MIC	MBC	MIC	MBC
*Staphylococcus aureus*	0.0625	0.25	0.25	0.25	0.25	0.25	0.25	0.25	0.25	0.25
*Streptococcus pyogenes*	0.0625	0.125	0.125	0.25	0.125	0.25	0.125	0.25	0.125	0.25
*Escherichia coli*	0.0078	0.0078	0.0078	0.031	0.0078	0.031	0.0078	0.031	0.0078	0.015
*Pseudomonas aeruginosa*	0.5	1	1	2	1	2	1	2	1	2

### Haemolysis of erythrocytes

**Fig. 6:**
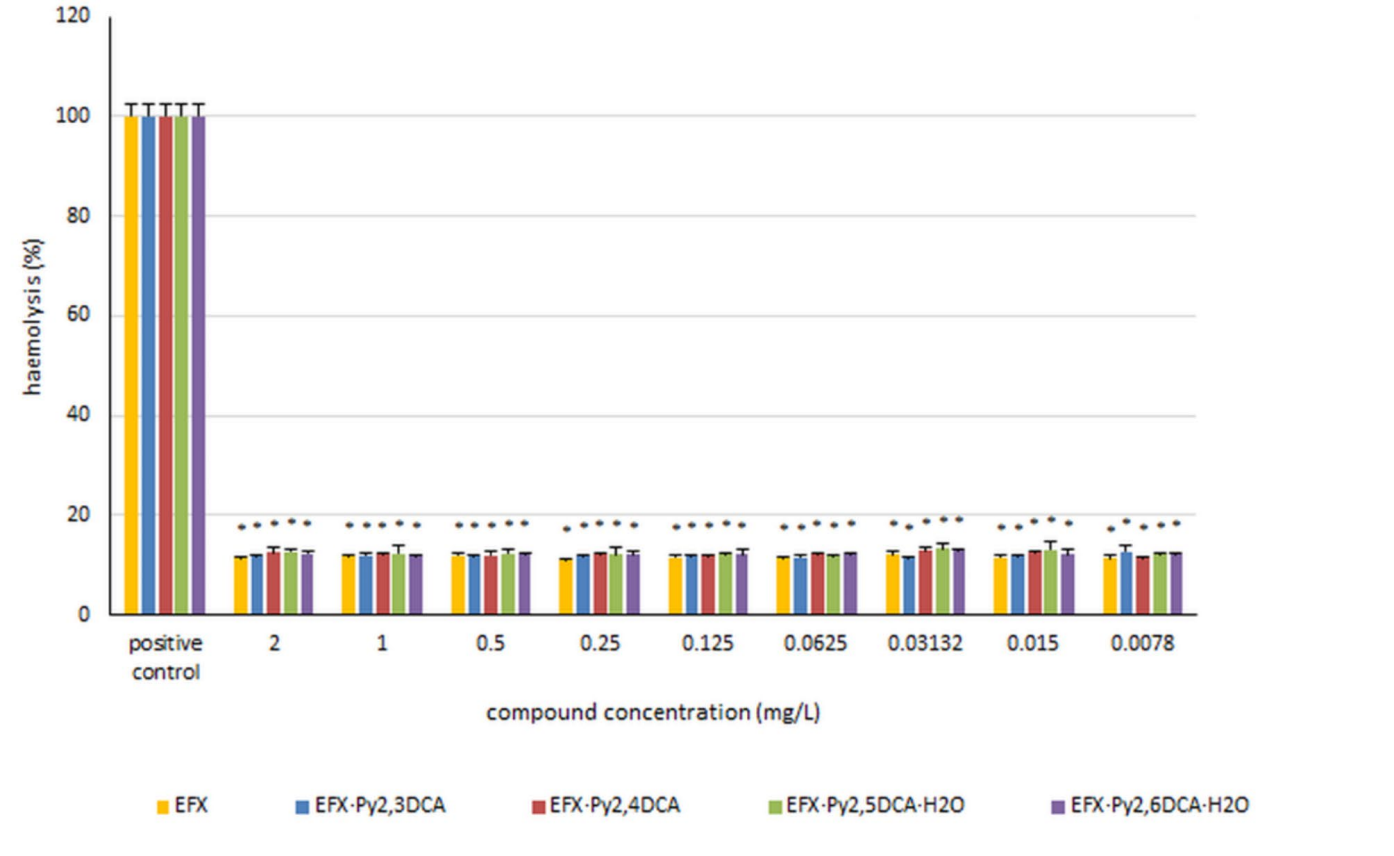
The haemolysis of erythrocytes in the presence of the tested compounds. The obtained results are expressed as the mean ± SD. * - represents statistically-significant results (*p* ≤ 0.05) determined via one-way analysis of variance (ANOVA).

The potential of pure enrofloxacin and its new salts with pyridinedicarboxylic acids to cause haemolysis was calculated after 24-hour incubation with red blood cells. The findings (Fig. 6) indicate that the tested compounds exhibit negligible haemolytic activity amounting to approximately 10%. Neither enrofloxacin nor the analysed salts had any significant effect on the red blood cells.

## Methods

### Synthesis and crystallization

All reagents were obtained commercially and were used without further purification. Enrofloxacin (purity 98%) was purchased from Angene Chemical Private Limited (India), while pyridine-2,4-dicarboxylic acid (purity 99%), pyridine-2,5-dicarboxylic acid (purity 99%) was purchased from Fluorochem (United Kingdom), and pyridine-2,3-dicarboxylic acid (purity 99%), pyridine-2,6-dicarboxylic acid (purity 99%) were purchased from Sigma-Aldrich Chemie (Germany).

For salt syntheses, equimolar quantities (0.05 mmol of each component) of enrofloxacin and pyridine-2,3-dicarboxylic acid / pyridine-2,4-dicarboxylic acid / pyridine-2,5-dicarboxylic acid / pyridine-2,6-dicarboxylic acid, respectively, were ground together in a mortar and pestle. The resulting fine powder mixtures were dissolved in a solution of methanol and ethanol (1:1 v/v) with a drop of acetone for EFX·Py2,5DCA·H_2_O, or a solution of toluene, methanol and ethanol in a 2:1:1 volume ratio for EFX·Py2,3DCA, EFX·Py2,4DCA and EFX·Py2,6DCA·H_2_O. All solutions were heated to 72 °C. The resulting solutions were filtered and covered by perforated paraffin film. Finally, the filtrates were evaporated slowly at 4 °C in the refrigerator until crystals appeared.

### Single-crystal X-ray diffraction

Crystal data, data collection and structure refinement details are summarized in Table 2. Single-crystal X-ray experiments were carried out with Cu*K*_α_ radiation using Rigaku XtaLab Synergy diffractometer equipped with a HyPix detector. The structures were solved by dual-space methods (SHELXT^50^) and refined by full-matrix least-squares procedures (SHELXL^51^).

**Table 2:** Crystal data, data collection and structure refinement details for enrofloxacin salts.

	EFX·Py2,3DCA	EFX·Py2,4DCA	EFX·Py2,5DCA·H_2_O	EFX·Py2,6DCA·H_2_O
CCDC number	2,363,588	2,363,589	2,363,590	2,363,591
Crystal data				
Chemical formula	C_19_H_23_FN_3_O_3_·C_7_H_4_NO_4_	C_19_H_23_FN_3_O_3_·C_7_H_4_NO_4_	C_7_H_4_NO_4_·C_19_H_23_FN_3_O_3_·H_2_O	C_19_H_23_FN_3_O_3_·C_7_H_4_NO_4_·H_2_O
*M*_r_	526.51	526.51	544.53	544.53
Crystal system, space group	Monoclinic, *P*2_1_/*c*	Triclinic, *P*$\:\stackrel{-}{1}$	Triclinic, *P*$\:\stackrel{-}{1}$	Triclinic, *P*$\:\stackrel{-}{1}$
Temperature (K)	100	293	293	293
*a*, *b*, *c* (Å)	15.4059 (3), 12.0237 (2), 14.1742 (2)	11.7939 (3), 13.1898 (4), 16.9342 (4)	9.4265 (2), 11.8496 (3), 12.8465 (3)	7.3943 (1), 9.4045 (1), 18.9718 (1)
α, β, γ (°)	90, 114.373 (2), 90	83.736 (2), 75.154 (2), 75.414 (2)	111.743 (2), 95.353 (2), 105.711 (2)	82.612 (1), 87.534 (1), 70.802 (1)
*V* (Å^3^)	2391.57 (8)	2461.47 (12)	1252.63 (5)	1235.58 (2)
*Z*	4	4	2	2
Radiation type	Cu *K*α	Cu *K*α	Cu *K*α	Cu *K*α
µ (mm^− 1^)	0.95	0.92	0.96	0.97
Crystal size (mm)	0.13 × 0.08 × 0.03	0.17 × 0.10 × 0.06	0.12 × 0.07 × 0.05	0.22 × 0.13 × 0.07
Data collection				
Diffractometer	XtaLAB Synergy, Dualflex, HyPix	XtaLAB Synergy, Dualflex, HyPix	XtaLAB Synergy, Dualflex, HyPix	XtaLAB Synergy, Dualflex, HyPix
Absorption correction	Gaussian *CrysAlis PRO*	Gaussian *CrysAlis PRO*	Gaussian *CrysAlis PRO*	Gaussian *CrysAlis PRO*
*T*_min_, *T*_max_	0.755, 1.000	0.812, 1.000	0.895, 1.000	0.689, 1.000
No. of measured, independent and observed [*I* > 2σ(*I*)] reflections	30,496, 4804, 4445	29,600, 9503, 7655	13,372, 4553, 3758	42,442, 4733, 4376
*R*_int_	0.030	0.029	0.033	0.022
(sin θ/λ)_max_ (Å^−1^)	0.625	0.617	0.602	0.617
Refinement				
*R*[*F*^2^ > 2σ(*F*^2^)], *wR*(*F*^2^), *S*	0.037, 0.098, 1.07	0.053, 0.150, 1.04	0.047, 0.135, 1.07	0.034, 0.095, 1.04
No. of reflections, parameters and restraints	4804, 356, 3	9503, 711, 2	4553, 366, 2	4733, 373, 1
Δρ_max_, Δρ_min_ (e Å^−3^)	0.27, − 0.29	0.44, − 0.43	0.33, − 0.23	0.23, − 0.17

The H atoms on the C atoms were placed geometrically and refined using a riding model with isotropic displacement parameters equal to *U*_iso_(H) = 1.2 *U*_eq_(C) for the methylene, methine and aromatic groups, and 1.5 *U*_eq_(C) of the attached C atom for methyl groups.

The hydrogen atoms bonded to heteroatoms (N, O), involved in hydrogen bonds, were located in difference Fourier maps and refined freely. Distance restraints (O−H = 0.82Å and N−H = 0.87Å) were occasionally applied. In EFX·Py2,6DCA·H_2_O, the riding model for the water molecule was used (AFIX 6) and *U*_iso_(H) = 1.5 *U*_eq_(O).

### FT-IR

The Fourier-transform infrared (FT-IR) spectra of pure enrofloxacin and four enrofloxacin salts were collected using a Thermo Nicolet Nexus FT-IR in the range of wave numbers from 4000 cm^−1^ to 400 cm^−1^ in the solid state with KBr pellets. Data were analysed using OMNIC software.

### Thermogravimetric analysis

The four enrofloxacin salts were subjected to thermogravimetric measurements (TGA) with the Netzsch TG 209F1 Iris and Netzsch STA 449 F1 Jupiter; the latter was additionally coupled with an Aeolos Quadro QMS 403 mass spectrometer for supportive interpretation. Samples were heated in ceramic crucibles to 800 °C or/and 500 °C, at a heating rate of 10 °C min^− 1^ in atmospheric air. The experimental data were processed using NETZSCH Proteus software.

### Water solubility

The solubility of enrofloxacin salts in water was measured by the gravimetric method at a temperature of 25 °C. Temperature was maintained using a calibrated UltraUB 20 F with a DLK 25 circulating cooler (Lauda, Germany). A specific amount of the substance under investigation was carefully weighed using a precise analytical balance (Sartorius RC 210D). The mixture of salts and water was thoroughly mixed to ensure complete dissolution of the substance under investigation and left for 2 h to achieve equilibrium between the solution and the precipitate. Then, the solution was filtered, and the remaining precipitate was dried to a constant mass to remove the solvent. The difference between the initial mass of the substance m_0_ and the mass of the undissolved substance m_1_ allowed for the determination of the mass of the substance dissolved in the given mass of solvent.

Each measurement was taken in triplicate.

### Assessment of antibacterial properties

The antibacterial activity of the enrofloxacin salts was determined against two Gram-positive strains (*Staphylococcus aureus* ATCC 6358, *Streptococcus pyogenes* ATCC 19615) and two Gram-negative bacteria (*Escherichia coli* ATCC 25922, *Pseudomonas aeruginosa* ATCC 15442). The determination was performed in accordance with Clinical and Laboratory Standards Institute (CLSI) standard M07 for antimicrobial susceptibility testing of aerobic bacteria.

The assessment was performed using the broth microdilution method with Mueller-Hinton medium. The tested samples were accompanied by biotic controls, i.e. without the addition of newly synthesized compounds, and abiotic controls, i.e. without the addition of microorganisms. All probes were incubated for 24 h at 37 °C. Microbial growth was measured using a microplate reader (Multiskan FC Microplate Photometer, ThermoFisher Scientific, Pudong, Shanghai, China) at λ = 630 nm. The follow were determined: the minimal inhibitory concentration (MIC), defined as the lowest concentration of a compound that prevents visible growth of bacteria, and minimal bactericidal concentration (MBC), defined as the lowest concentration of a substance that completely limits the viability of microorganisms.

### Determination of haemolytic activity

The pure enrofloxacin and its salts were subjected to cytotoxicity testing with red blood cells from the Lodz Regional Center of Blood Donation and Blood Treatment (Poland). The erythrocytes used in the study were initially washed three times with PBS; they were then diluted in buffer containing the tested compounds so that the haematocrit was 2.5%. At the same time, negative controls containing PBS and positive controls containing water were prepared. All samples were incubated in the dark at 37 °C for 24 h. Following this, they were centrifuged and the degree of haemolysis determined spectrophotometrically at a wavelength of λ = 540 nm using a MultiskanTM FC Microplate Photometer (Thermo Fisher Scientific, Pudong, Shanghai, China). The haemolytic activity of the tested enrofloxacin salts with isomeric pyridinedicarboxylic acids was determined using the following formula:

$$\% Haemolysis=As/Ac \times 100\% $$

Where, As – is the absorbance of samples incubated with substance; Ac – is the absorbance of the samples containing red blood cells suspended in water.

All biological experiments were carried out in duplicate and the results are presented as the mean values of three trials (*n* = 3) of each experiment with standard deviation.

## Conclusion

The present work describes the successful crystallization of the salts of enrofloxacin with four isomeric pyridine-2,n-dicarboxylic acids (*n* = 3,4,5,6), EFX·Py2,3DCA, EFX·Py2,4DCA, EFX·Py2,5DCA·H_2_O and EFX·Py2,6DCA·H_2_O; two of which are monohydrates. It also presents their single-crystal structures. In all cases, proton transfer was confirmed from the acid to a nitrogen atom of the piperazine moiety; however, only three salts demonstrated the formation of a cation – anion pair. In EFX·Py2,5DCA·H_2_O, the enrofloxacin cation interacts directly with a water molecule, which is an acceptor of the N3−H3*A*···O1*W* hydrogen bond. Another interesting feature of this crystal structure is the presence of mono-periodic polymeric chains built from anions; this is made possible by the presence of two hydrogen atoms shared equally between neighbouring carboxylate groups.

Compared to pure enrofloxacin, the EFX·Py2,5DCA·H_2_O salt demonstrated the least solubility in water (i.e. approximately a 17-fold increase), while EFX·Py2,4DCA showed the most significant increase in solubility (i.e. approximately a 115-fold increase).

The newly-synthesized enrofloxacin salts with isomeric pyridinedicarboxylic acids exhibit antibacterial properties against both Gram-positive and Gram-negative bacteria. They all offer greater or at least comparable effectiveness in relation to pure enrofloxacin. Furthermore, cytotoxicity tests performed using erythrocytes showed that tested enrofloxacin salts do not cause haemolysis of red blood cells at concentrations that effectively limit the growth of bacteria, which is a good prognosis for their potential use.

The new salts hence demonstrate greater solubility in water and enhanced antibacterial activity compared to the parent drug, enrofloxacin. As such, isomeric pyridine-2,n-dicarboxylic acids (*n* = 3,4,5,6) appear to be suitable cocrystallizing agents for improving the efficacy of pharmaceutical ingredients.

## Electronic supplementary material

Below is the link to the electronic supplementary material.

Supplementary Material 1

## Data Availability

Deposition numbers CCDC 2363588-2363591 contain the supplementary crystallographic data for the structures reported in this article. These data can be obtained free of charge from The Cambridge Crystallographic Data Centre via www.ccdc.cam.ac.uk/structures.
