# Supplementary material for: Enhancing the solubility and antibacterial activity of novel molecular salts of enrofloxacin drug with isomeric pyridinedicarboxylic acids
Source: Sci Rep. 2024 Nov 26;14:29317. doi: 10.1038/s41598-024-80665-y (PMC11599901; doi:10.1038/s41598-024-80665-y)
Supplement: Supplementary file 1 — Supplementary Material 1 [file 41598_2024_80665_MOESM1_ESM.pdf]

SUPPLEMENTARY INFORMATION

Enhancing the solubility and antibacterial activity of novel molecular salts of enrofloxacin drug with isomeric pyridinedicarboxylic acids

Valeryia Hushcha,[a] Anna Ben,[a,b] Aleksandra Felczak,[c*] Katarzyna Lisowska,[c]

Zdzisław Kinart,[a] Michał Gacki,[d] Lilianna Chęcińska[a*]

[a] Faculty of Chemistry, University of Lodz, Pomorska 163/165, 90-236 Lodz, Poland;

[b] University of Lodz Doctoral School of Exact and Natural Sciences, Narutowicza 68, 901-136 Lodz, Poland;

[c] Faculty of Biology and Environmental Protection, University of Lodz, Banacha 12/16, 90-237 Lodz, Poland;

[d] Institute of General and Ecological Chemistry, Faculty of Chemistry, Lodz University of Technology, Żeromskiego 116, 90-924 Lodz, Poland.

* corresponding authors

email address: lilianna.checinska@chemia.uni.lodz.pl and aleksandra.felczak@biol.uni.lodz.pl

Table of contents

	Figures and tables	Page
Table S1	Dihedral angles between the best planes of the selected rings of the enrofloxacin molecule in the analysed salts	2
Figure S1	An overlay of five independent enrofloxacin molecules	2
Table S2	Cremer & Pople puckering parameters for the piperazine ring of the enrofloxacin molecule in the analysed salts	2
Table S3	The angle between the N2–C7 (and N6–C37) bond and the normal to the Cremer & Pople mean plane of the piperazine ring (determining the site in the piperazine ring occupied by the quinolone ring) of the enrofloxacin molecule in the analysed salts	3
Table S4	Normalized parameters of the intramolecular C–H···F hydrogen-bond in the enrofloxacin molecule in the analysed salts	3
Table S5	Normalized parameters of the intramolecular O–H···O hydrogen-bond in the enrofloxacin and acid molecules in the analysed salts	3
Table S6	Hydrogen bond geometry for enrofloxacin salts	4
Table S7	Bond lengths within the carboxylic/carboxylate groups in acid molecules in the analysed salts	5
Figure S2	Supramolecular architectures of analysed enrofloxacin salts	6
Table S8	Geometric parameters of aromatic π-π interactions in the analysed salts	7
Figure S3	Fourier-transform infrared spectrum of enrofloxacin	8
Figure S4	Fourier-transform infrared spectra of EFX, Py2,3DCA and EFX·Py2,3DCA salt	8
Figure S5	Fourier-transform infrared spectra of EFX, Py2,4DCA and EFX·Py2,4DCA salt	9
Figure S6	Fourier-transform infrared spectra of EFX, Py2,5DCA and EFX·Py2,5DCA·H_2O salt	9
Figure S7	Fourier-transform infrared spectra of EFX, Py2,6DCA and EFX·Py2,6DCA·H_2O salt	10
Figure S8	Thermal decomposition of analysed enrofloxacin salts with pyridinedicarboxylic acids (TG, DTG and DTA curves)	10

Table S1. Dihedral angles (°) between the best planes of the selected rings of the enrofloxacin molecule in the analysed salts

1 – heterocyclic ring; 2 – benzene ring; 3 - piperazine ring

Structure	1/2	2/3
EFX·Py2,3DCA	1.34(6)	27.40(6)
EFX·Py2,4DCA	3.76(9)	51.56(10)
	2.60(11)	51.14(13)
EFX·Py2,5DCA·H_2O	1.98(10)	28.97(11)
EFX·Py2,6DCA·H_2O	2.23(6)	70.50(6)

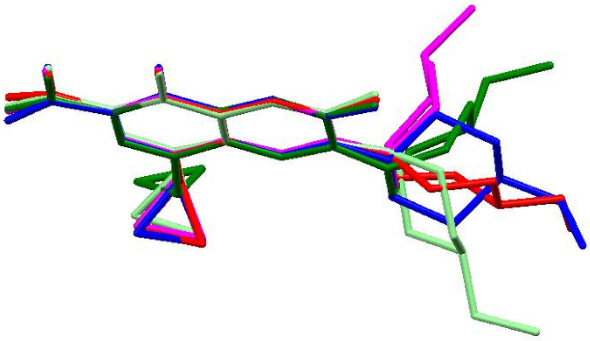

Figure S1. An overlay of five independent enrofloxacin molecules, showing the best fit for quinolone moiety: colour code is red = EFX(·Py2,3DCA); light green – A-EFX(·Py2,4DCA), ; dark green – B-EFX(·Py2,4DCA), blue – EFX(·Py2,5DCA·H_2O) and magenta – EFX(·Py2,6DCA·H_2O).

Table S2. Cremer & Pople puckering parameters (Å, °) for the piperazine ring of the enrofloxacin molecule in the analysed salts

Structure	Q	θ	φ
EFX·Py2,3DCA	0.582(1)	173.2(2)	243(1)
EFX·Py2,4DCA	0.561(2)	3.5(2)	241(3)
	0.593(3)	172.6(3)	141(2)
EFX·Py2,5DCA·H_2O	0.592(2)	2.8(2)	77(4)
EFX·Py2,6DCA·H_2O	0.561(1)	176.8(1)	195(2)

Table S3. The angle (°) between the N2–C7 (and N6–C37) bond and the normal to the Cremer & Pople mean plane of the piperazine ring (determining the site in the piperazine ring occupied by the quinolone ring) of the enrofloxacin molecule in the analysed salts

Structure	Angle	Position
EFX·Py2,3DCA	86.66(8)	equatorial
EFX·Py2,4DCA	39.13(14)	bisectional
	33.05(16)	bisectional
EFX·Py2,5DCA·H_2O	87.10(11)	equatorial
EFX·Py2,6DCA·H_2O	21.27(7)	axial

Table S4. Normalized parameters (Å, °) of the intramolecular C–H···F hydrogen-bond in the enrofloxacin molecule in the analysed salts

Structure	*D*—H···*A*	*D*—H	H···*A*	*D*—H···*A*
EFX·Py2,3DCA	C14–H14B···F1	1.10	2.12	123
EFX·Py2,4DCA	C14–H14B···F1	1.10	2.07	121
	C44–H44B···F2	1.10	1.97	129
EFX·Py2,5DCA·H_2O	C14–H14A···F1	1.10	2.13	123
EFX·Py2,6DCA·H_2O	C14–H14B···F1	1.10	2.09	124

Table S5. Normalized parameters (Å, °) of the intramolecular O–H···O hydrogen-bond in the enrofloxacin and acid molecules in the analysed salts

Structure	*D*—H···*A*	*D*—H	H···*A*	*D*—H···*A*
EFX·Py2,3DCA	O2–H2*A*···O1	0.98	1.55	159
	O6–H6*A*···O4	0.98	1.42	171
EFX·Py2,4DCA	O2–H2*A*···O1	0.98	1.61	153
	O9–H9*A*···O8	0.98	1.62	149
EFX·Py2,5DCA·H_2O	O2–H2*A*···O1	0.98	1.60	155
EFX·Py2,6DCA·H_2O	O2–H2*A*···O1	0.98	1.64	154

Table S6. Hydrogen bond geometry (Å, °) for enrofloxacin salts

D–H···A	D–H	H···A	D···A	D–H···A
EFX·Py2,3DCA				
O2–H2A···O1	0.87 (2)	1.66 (2)	2.4960 (13)	160 (2)
N3–H3A···O5	0.89 (1)	1.90 (2)	2.6872 (15)	146 (2)
N3–H3A···N4	0.89 (1)	2.42 (2)	3.1488 (16)	140 (2)
O6–H6A···O4	0.86 (1)	1.55 (1)	2.3988 (14)	171 (3)
C5–H5···O4^{i}	0.95	2.50	3.4242 (16)	165
C11–H11···O6^{ii}	1.00	2.36	3.2134 (17)	143
C14–H14B···F1	0.99	2.18	2.8647 (15)	125
C15–H15B···O3^{iii}	0.99	2.42	3.1555 (16)	130
C16–H16B···O2^{iv}	0.99	2.32	3.1873 (16)	146
EFX·Py2,4DCA				
O2–H2A···O1	0.87 (3)	1.71 (3)	2.523 (2)	155 (3)
N3–H3A···O4	0.90 (2)	1.94 (2)	2.836 (2)	178 (2)
O9–H9A···O8	0.92 (4)	1.68 (4)	2.515 (3)	151 (3)
N7–H7A···O12	0.90 (3)	1.93 (3)	2.817 (3)	168 (2)
O6–H6···O12^{i}	0.87 (2)	1.65 (2)	2.518 (2)	173 (3)
O14–H14···O4	0.86 (2)	1.66 (2)	2.5157 (19)	169 (3)
C11–H11···O5^{ii}	0.98	2.49	3.253 (2)	135
C12–H12B···O1^{iii}	0.97	2.40	3.343 (3)	163
C14–H14A···F1	0.97	2.14	2.794 (2)	124
C15–H15A···O3^{iv}	0.97	2.28	3.159 (3)	150
C18–H18A···O13	0.97	2.51	3.198 (3)	128
C42–H42B···O8^{v}	0.97	2.43	3.375 (4)	166
C44–H44B···F2	0.97	2.06	2.803 (4)	132
C45–H45A···O10^{v}	0.97	2.47	3.436 (4)	172
C45–H45B···O10^{vi}	0.97	2.34	3.269 (3)	160
C47–H47A···O7^{vii}	0.97	2.52	3.251 (4)	132
C48–H48A···O1^{ii}	0.97	2.43	3.350 (4)	159
EFX·Py2,5DCA·H_2O				
O2–H2A···O1	0.88 (2)	1.69 (2)	2.523 (2)	156 (3)
N3–H3A···O1W	0.91 (2)	1.76 (2)	2.663 (3)	169 (3)
O4–H4A···O4^{i}	1.23	1.23	2.449 (2)	180
O6–H6A···O6^{ii}	1.23	1.23	2.456 (2)	180
O1W–H1W1···O7	0.86	1.88	2.7082 (19)	161
O1W–H2W1···N4^{iii}	0.87	2.02	2.867 (2)	163
C14–H14A···F1	0.97	2.20	2.871 (2)	125
C15–H15A···O3^{iv}	0.97	2.52	3.286 (2)	136
C16–H16A···O2^{v}	0.97	2.45	3.295 (3)	145
C17–H17A···O4^{iii}	0.97	2.49	3.440 (3)	166
EFX·Py2,6DCA·H_2O				
O2—H2A···O1	0.90 (2)	1.71 (2)	2.5571 (14)	155 (2)
N3—H3A···O4	0.936 (18)	1.696 (18)	2.6133 (13)	165.6 (15)
O6—H6A···O1W	0.90 (2)	1.79 (2)	2.6637 (16)	163.3 (18)
O1W—H1W1···O5^{i}	0.85 (2)	1.94 (2)	2.7714 (16)	169 (2)
O1W—H2W1···O5	0.90 (2)	2.00 (2)	2.8876 (16)	171 (2)
C12—H12A···O1^{ii}	0.97	2.37	3.3012 (17)	162
C14—H14B···F1	0.97	2.17	2.8577 (14)	127
C15—H15A···O3^{iii}	0.97	2.38	3.3405 (15)	172

Symmetry codes: EFX·Py2,3DCA (i) −*x*+1, *y*−1/2, −*z*+1/2; (ii) *x*, −*y*+3/2, *z*−1/2; (iii) *x*, *y*+1, *z*; (iv) −*x*+1, −*y*+1, −*z*; EFX·Py2,4DCA (i) *x*−1, *y*, *z*+1; (ii) −*x*+1, −*y*+2, −*z*+1; (iii) −*x*, −*y*+3, −*z*+1; (iv) *x*, *y*−1, *z*; (v) −*x*+3, −*y*, −*z*; (vi) *x*, *y*+1, *z*; (vii) −*x*+1, −*y*+1, −*z*+1; EFX·Py2,5DCA·H_2O (i) −*x*+1, −*y*−1, −*z*−1; (ii) −*x*, −*y*, −*z*; (iii) −*x*+1, −*y*, −*z*; (iv) *x*−1, *y*−1, *z*−1; (v) −*x*+1, −*y*+2, −*z*+1; EFX·Py2,6DCA·H_2O (i) −*x*+1, −*y*+1, −*z*+1; (ii) −*x*+1, −*y*, −*z*+2; (iii) −*x*, −*y*, −*z*+2.

Table S7. Bond lengths (Å) within the carboxylic/carboxylate groups in acid molecules in the analysed salts

Structure	single C–O	double C=O	single C–O	double C=O
	C25–O4(-)<br>O55–C12(-)	C25–O25<br>C55–O11	C26–O6(H)<br>C56–O14(H)	C26–O7<br>C56–O13
EFX·Py2,3DCA	1.2824(2)	1.2361(2)	1.2952(2)	1.2248(2)
EFX·Py2,4DCA	1.290(2)<br>1.278(3)	1.221(2)<br>1.223(3)	1.292(3)<br>1.305(2)	1.210(3)<br>1.212(2)
EFX·Py2,5DCA·H_2O	1.279(2)	1.222(2)	1.275(2)	1.217(2)
EFX·Py2,6DCA·H_2O	1.2468(2)	1.2354(2)	1.3195(2)	1.2094(2)

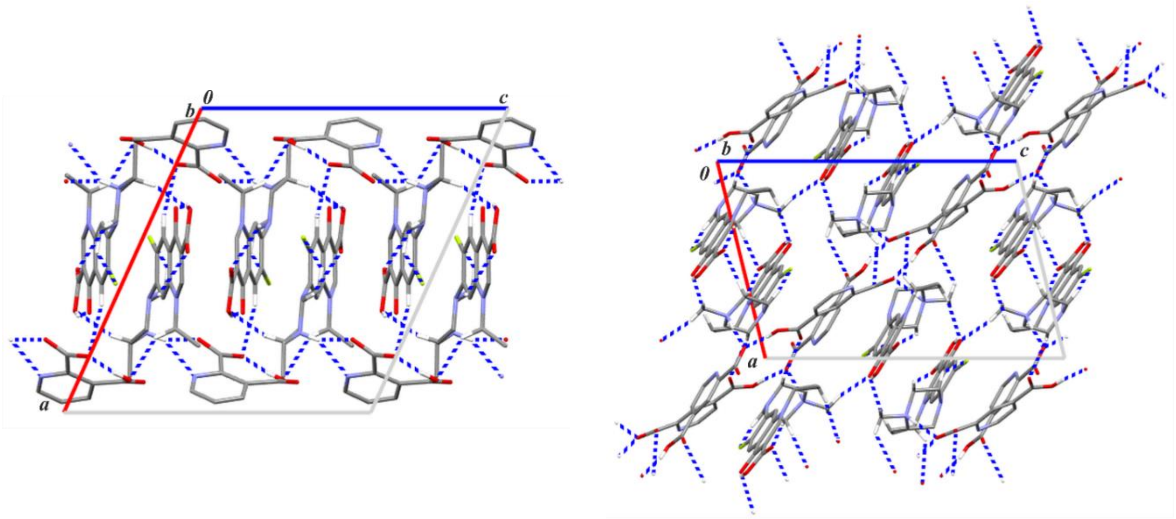

(a) (b)

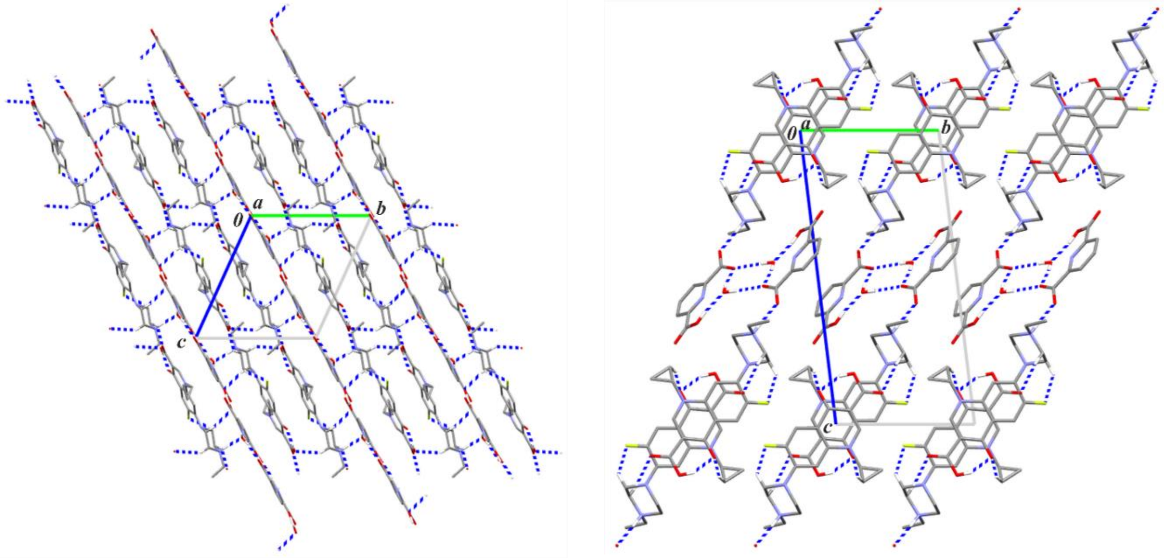

(c) (d)

Figure S2. Supramolecular architectures of EFX·Py2,3DCA (a), EFX·Py2,4DCA (b), EFX·Py2,5DCA·H_2O and EFX·Py2,6DCA·H_2O (d); viewed down the crystallographic *b* axis (a-b) and *a* axis (c-d), respectively.

Table S8. Geometric parameters (Å, °) of aromatic π-π interactions in the analysed salts

1, 6 – heterocyclic ring; 2, 7 – benzene ring; 5, 10 - pyridine ring;

Structure	Interaction	*Cg*(I)···*Cg*(J)	α	$Cg(I)_{perp}$	$Cg(J)_{perp}$	Slippage
EFX·Py2,3DCA	*Cg*(1) *Cg*(2)^{iv}	3.4755(7)	1.34(6)	3.3370(5)	3.3346(5)	0.979
	Cg(5) *Cg*(5)^{v}	4.0219(9)	0.02(7)	3.6615(6)	3.6615(6)	1.664
EFX·Py2,4DCA	*Cg*(1)···*Cg*(1)^{iii}	3.7786(12)	0.00(9)	3.5109(8)	3.5109(8)	1.397
	Cg(1)···*Cg*(2)^{iii}	3.9801(11)	3.76(9)	3.5002(8)	3.5838(8)	1.731
	Cg(2)···*Cg*(10)^{ii}	3.6456(11)	5.03(10)	3.3942(8)	3.4235(8)	1.253
	Cg(6)···*Cg*(6)^{v}	4.0361(14)	0.02(11)	3.5985(10)	3.5986(10)	1.238
	Cg(6)···*Cg*(7)^{v}	4.0361(14)	2.60(11)	3.5716(10)	3.6432(10)	1.737
EFX·Py2,5DCA·H_2O	*Cg*(1)···*Cg*(2)^{v}	3.5682(12)	1.98(10)	3.4405(8)	3.4712(8)	0.826
	Cg(1)···*Cg*(5)^{vi}	4.0184(12)	8.58(10)	3.3951(8)	3.6604(8)	1.658
	Cg(2)···*Cg*(5)^{vi}	4.0419(11)	10.46(10)	3.5523(8)	3.6829(8)	1.665
EFX·Py2,6DCA·H_2O	*Cg*(1)···*Cg*(1)^{iii}	3.6599(7)	0.00(6)	3.5379(5)	3.5379(5)	0.937
	Cg(1)···*Cg*(2)^{ii}	4.0550(7)	2.23(6)	3.6231(5)	3.6859(5)	1.690
	Cg(5)···*Cg*(5)^{iv}	3.6509(7)	0.00(6)	3.4076(5)	3.4076(5)	1.310

Symmetry codes: EFX·Py2,3DCA (iv) −*x*+1, −*y*+1, −*z*; (v) −*x*+2, *y*−+2, −*z*+1; EFX·Py2,4DCA (ii) −*x*+1, −*y*+2, −*z*+1; (iii) −*x*, −*y*+3, −*z*+1; (v) −*x*+3, −*y*, −*z*; EFX·Py2,5DCA·H_2O (v) ,−*x*+1, −*y*+2, −*z*+1; (vi) *x*, *y*+1, *z*+1; EFX·Py2,6DCA·H_2O (ii) −*x*+1, −*y*, −*z*+2; (iii) −*x*, −*y*, −*z*+2; (iv) −*x*, −*y*+2, −*z*+1.

Cg(I)···*Cg*(J) – distance between ring centroids; α - dihedral angle between planes I and J; $Cg(I)_{perp}$ and $Cg(J)_{perp}$ - (interplanar spacing) perpendicular distance of *Cg*(I) on ring J and *Cg*(J) on ring I, respectively; slippage - distance between *Cg*(I) and perpendicular projection of *Cg*(J) on ring I.

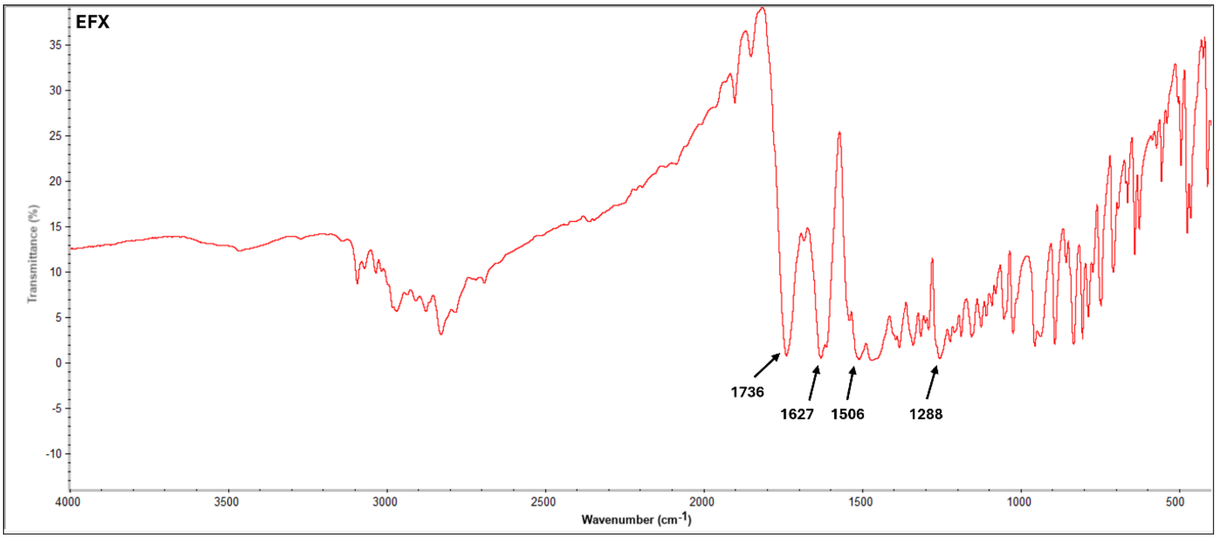

Figure S3. Fourier-transform infrared spectrum of enrofloxacin (EFX).

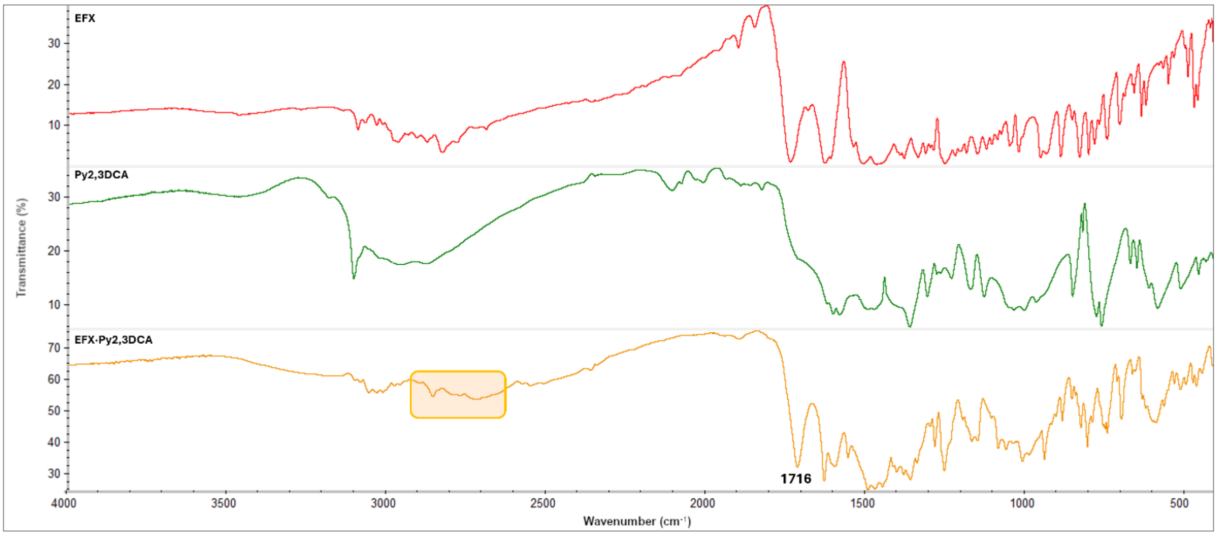

Figure S4. Fourier-transform infrared spectra of enrofloxacin (EFX) (red), pyridine-2,3-dicarboxylic acid (Py2,3DCA) (green), and EFX·Py2,3DCA salt (yellow).

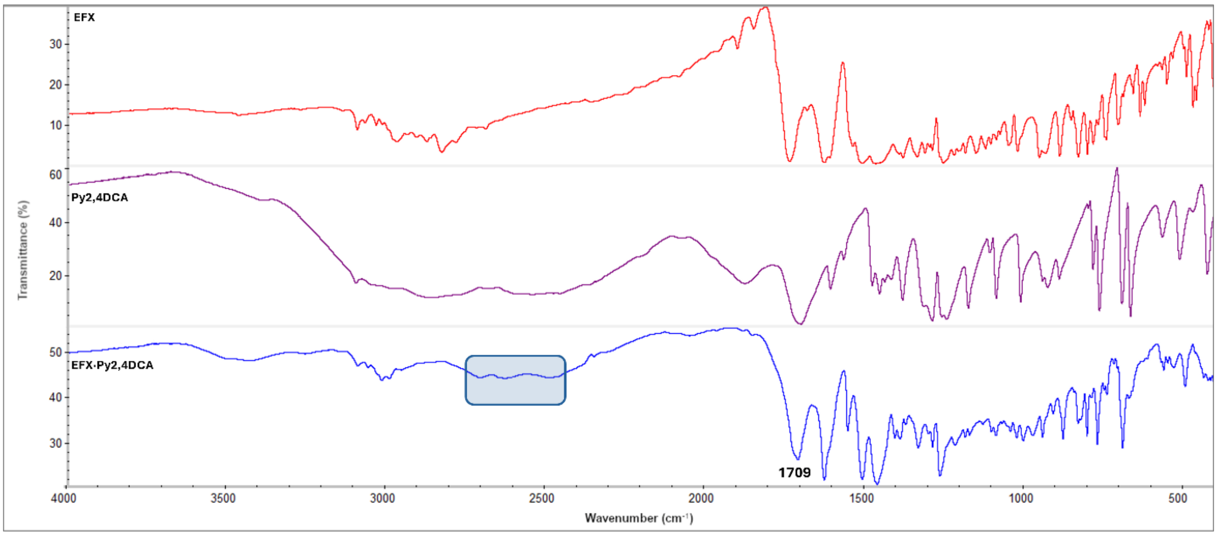

Figure S5. Fourier-transform infrared spectra of enrofloxacin (EFX) (red), pyridine-2,4-dicarboxylic acid (Py2,4DCA) (violet), and EFX·Py2,4DCA salt (blue).

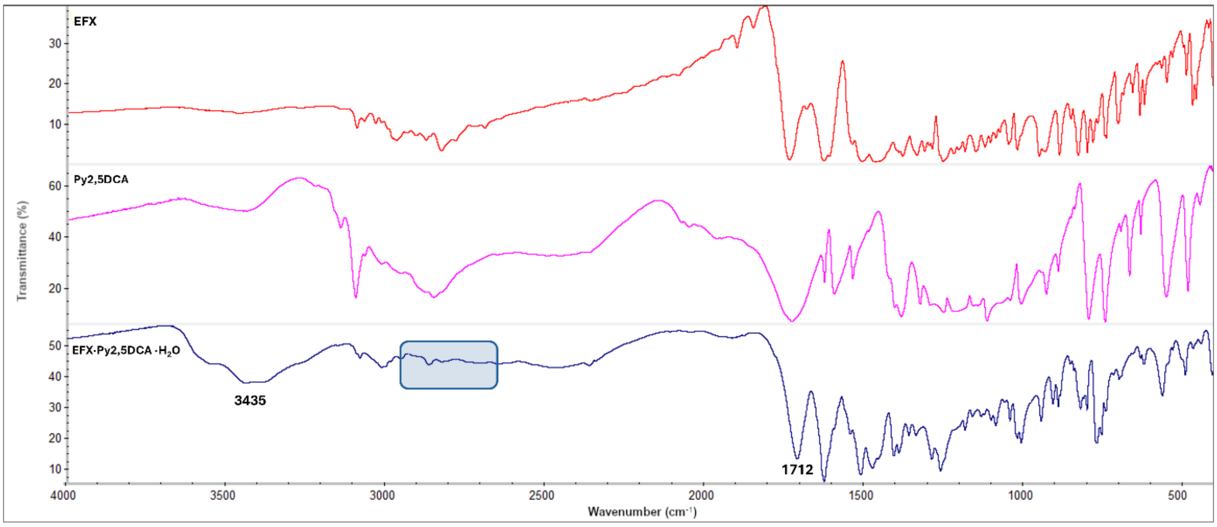

Figure S6. Fourier-transform infrared spectra of enrofloxacin (EFX) (red), pyridine-2,5-dicarboxylic acid (Py2,5DCA) (magenta), and EFX·Py2,5DCA·H_2O salt (navy blue).

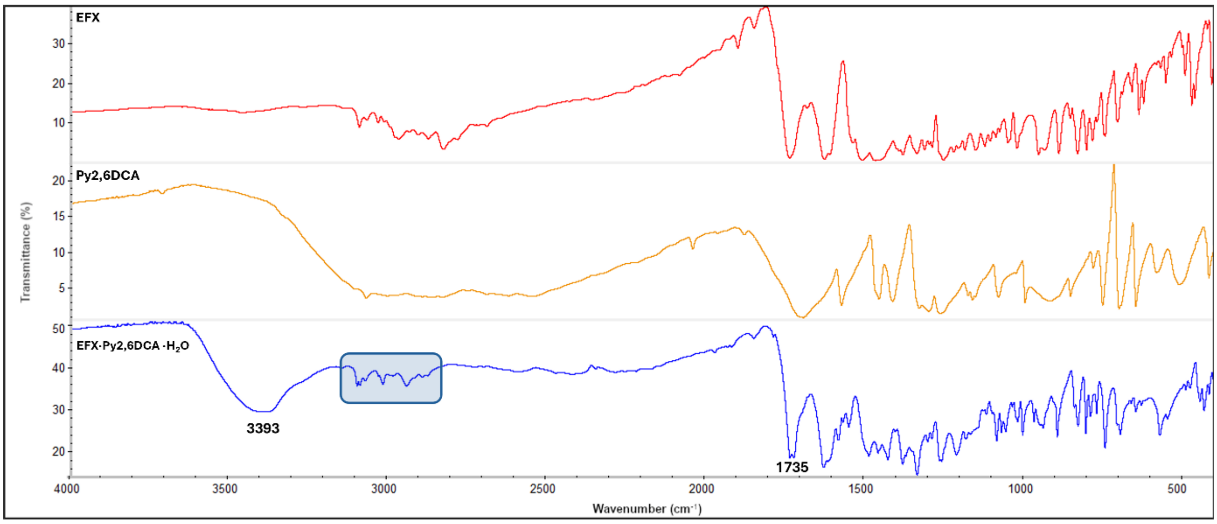

Figure S7. Fourier-transform infrared spectra of enrofloxacin (EFX) (red), pyridine-2,6-dicarboxylic acid (Py2,6DCA) (yellow), and EFX·Py2,6DCA·H_2O salt (blue).

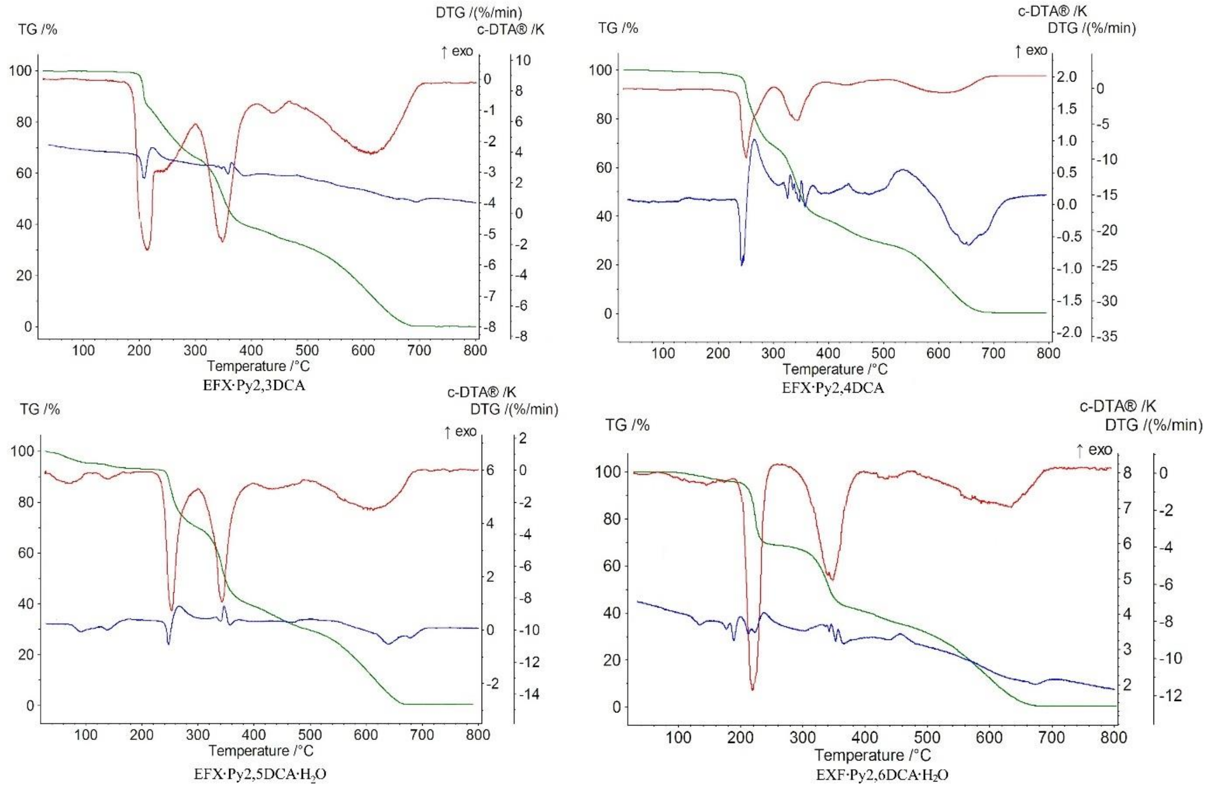

Figure S8. Thermal decomposition of analysed enrofloxacin salts with pyridinedicarboxylic acids. TG (thermogravimetric) curves are plotted in green, DTG (derivative thermogravimetric) curves are in red and DTA (differential thermal analysis) curves are in blue.
